# Unveiling the efficiency of pulsed electric field and ultrasonication in enhancing collagen susceptibility to enzymatic hydrolysis

**DOI:** 10.1016/j.ultsonch.2025.107633

**Published:** 2025-10-20

**Authors:** Elahe Sharifi, Ingrid Undeland, Mehdi Abdollahi

**Affiliations:** Department of Biology and Biological Engineering–Food and Nutrition Science, Chalmers University of Technology, 412 96 Gothenburg, Sweden

**Keywords:** Ultrasonication, Pulsed electric field, Collagen peptides, Antioxidation, Enzymatic hydrolysis

## Abstract

Since collagen has naturally evolved to be durable, producing collagen hydrolysate with low molecular weight (LMW) peptides which typically results in high bioactivity is a challenge. Here, the efficiency of ultrasound (US) and pulsed electric field (PEF) pretreatments in enhancing the enzymatic hydrolysis of starfish collagen biomass (SFCB) and the antioxidant capacity of the resulting hydrolysate were evaluated. Both pretreatments significantly improved the degree of hydrolysis (DH) and reduced the required hydrolysis time compared to the conventional methods. The DH of SFCB hydrolysate pretreated with US reached 20 % at 60 min while that of the PEF treatment reached 21 % at 120 min, matching the DH of the conventional hydrolysis at 240 min. US and PEF hereby increased the collagen hydrolysate recovery, particularly compensating for a low enzyme-to-substrate ratio (0.5 % v/w). The pretreatments resulted in collagen peptides with significantly higher antioxidant properties, as assessed by ABTS and DPPH assays. The IC50 values of ABTS radical inhibition were found to be 0.68, 0.43, and 0.84 mg/ml for the collagen peptides produced by the US, PEF pretreatments and conventional enzymatic hydrolysis, respectively. Applying PEF and US pretreatment at an enzyme concentration of 0.5 % v/w, increased the proportion of collagen hydrolysate with MW < 10 KDa to 64 % and 59 %, respectively, compared to the conventional method which yielded only 43 %. The enhancements in the antioxidant activities were linked to the increased proportion of LMW peptides (<10 kDa) and elevated relative levels of antioxidative amino acids e.g. glycine, proline, basic amino acids, and hydrophobic amino acids in the peptides produced using US and PEF pretreatment. Overall, US and PEF pretreatments effectively increased collagen susceptibility to enzymatic hydrolysis, improving efficiency and generating peptides with superior antioxidant properties.

## 1 Introduction

Collagen, a multifunctional macromolecule, accounts for 20–30 % of the total protein content in living organisms [1]. It has a unique amino acid composition, with glycine making up approximately 33 %, and proline together with hydroxyproline comprising around 22 % of its total amino acid content [2]. Due to its exceptional biological activities, native collagen is widely used in medical and food domains [[3], [4], [5]]. Collagen peptides derived from enzymatic hydrolysis can also exhibit antioxidant and anti-inflammatory properties as seen in some studys. Fro example, oral intake of hydrolyzed type 1 collagen demonstrated chondroprotective and anti-inflammatory effects in a murine osteoarthritis model [6]. Low molecular weight collagen peptides from Alaska pollock skin improved barrier function and reduced inflammation in Caco-2 cells [7]. Hydrolyzed fish collagen improved skin elasticity, collagen structure, and joint health over a 90 days period [8]. These findings highlight collagen peptides as a promsing natural ingredient for nutraceutical and nutricosmetic applications. The high abundance of proline in collagen, particularly in low molecular weight (LMW) peptides, can significantly enhance the protection of cells from oxidative damage caused by free radicals, as seen in an radical-induced in vitro cytotoxicity assay [9]. This makes collagen a very promising source for generating antioxidant peptides [10].

However, conventional enzymatic hydrolysis is less efficient when applied to collagen-rich tissues, especially those rich in collagen type I, than non-collagenous tissues, making the process more resource-intensive, tedious and laborious [10]. This inefficiency is mostly because collagen has naturally evoloved to be highly durable, relying on a triple-helix structure stabilized by extensive inter- and intramolecular hydrogen bonds and covalent crosslinkages which limits enzymes access to its peptide bonds and reduces hydrolysis efficiency [11]. A key strategy to boost the production of LMW-peptides from collagen with potential antioxidant activity is thus to first destabilize its structure and thereby promote its susceptibility to enzymatic hydrolysis [12]. Here, combining non-thermal assistant technologies with the enzymatic hydrolysis can be a promising solutions as suggested by previous studies [13].

Among non-thermal technologies, pulsed electric field (PEF) and ultrasound (US) treatments offer distinct advantages for enhancing collagen's susceptibility to enzymatic hydrolysis, potentially improving extraction efficiency and, ultimately, environmental sustainability of the peptide production process. PEF employs high-voltage, short electric pulses inducing electroporation, thereby facilitating efficient extraction of high value compounds [14,15]while substantially shortening processing times and reducing environmental impact. US technology, in contrast, uses sound waves to induce mechanical compression within cellular structures leading to cavitation. The cavitation effect creates localized shear forces and shock waves that disrupt biological tissues and accelerate heat and mass transfer. This enables a faster and more effective release of compounds which has made US particularly effective in native collagen extraction from fish by-products, achieving higher yields and shorter processing times than conventional methods[16,17].

Both PEF and US treatments thus show potential as pretreatments to improve the efficiency of collagen hydrolysis. Specifically, US treatment could unfold proteins through cavitation, enhancing enzyme-substrate accessability, which in turn can increase the degree of hydrolysis (DH) and facilitate the production of LMW-peptides, particularly at lower enzyme concentrations. For example, Xu et al. [18] reported that applying US on deer tandon collagen induced molecular unfolding and led to the disappearance of collagen secondary structure components which increased degree of hydrolysis. Ahmad et al. [19] observed similar effects on collagen sources such as bighead carp scales and bovine skin, attributed to cavitation-induced shear forces, microjets, and shock waves that break down collagen aggregates as was also eported for egg white proteins [20]. PEF, meanwhile, induces polarization of protein molecules at low intensities, unfolding protein structures and thus exposing hydrophobic amino acids. However, once the PEF intensity surpasses a specific threshold, thermal effects can however cause both denaturation and aggregation of heat-sensitive proteins e.g. whey [21,22]. PEF can also disrupt protein secondary structure, increasing β-sheets and decreasing α-helices as reported for whey protein [22,23]but this level of denaturation has not yet been studied in relation to collagen. While both PEF and US can induce protein denaturation and therbye enhance collagen’s susceptibility to enzymatic hydrolysis, they employ distinct mechanisms, which may lead to potentially different outcomes in terms of peptide molecular weight, amino acid composition, and thereby their antioxidant capacity. However, to the best of our knowledge, a side-by-side evaluation of PEF and US as pretreatments for enzymatic hydrolysis of marine collagen sources has not yet been reported, representing a critical gap in the research.

Starfish are an underutilized marine resource. Among various species, *Asterias* spp*.* poses significant threats to bivalve producers and is considered one of the most destructive invasive starfish species [24]. In northern Europe, the common starfish (*Asterias rubens*) negatively impacts biological and environmental systems and disrupts the mussel farming industry. Despite containing valuable biomolecules like collagen, these starfish are typically discarded during the harvesting of the mussels [25]. A few recent studies have addressed the extraction of native starfish collagen [26,27], but there is currently limited information about enzymatic generation of antioxidative collagen peptides from starfish; none using assisting technologies for enhanced enzymatic action.

The present study aimed to investigate the potential of innovative technologies, specifically PEF and US, as pretreatments for enhancing the susceptibility of starfish collagen to enzymatic hydrolysis, ultimately generating smaller peptides using less enzyme and time. The effect of both PEF and US at two different enzyme to substrate ratios (0.5 % and 1 % 0.5 and 1 % w/w of total protein in the samples) on DH, collagen hydrolysate yield, in vitro antioxidant activity, molecular weight distribution and amino acid composition of the generated collagen peptides were investigated.

## 2 Materials and methods

### 2.1 Materials

Common starfish (*Asterias rubens*) were obtained from Scanfjord Mollösund AB (Mollösund, Sweden), a mussel farming company located in Orust, Sweden. Starfish were mixed with ice and carefully transported to the laboratory. Subsequently, the starfish underwent a cleansing process utilizing chilled water within the laboratory, cut into pieces sized 2.5 × 2.5 cm, then packed in plastic bags and stored at 80 °C.

### 2.2 Chemicals

The chemicals and reagents used in this study were of scientific grade. Acetic acid, sodium hydroxide, hydrochloric acid and acetonitrile were supplied by Merck (Merck Life Sciences, Sweden). 2,2-diphenyl-1-picrylhydrazyl (DPPH), 2,2′-azino-bis(3-ethylbenzothiazoline-6-sulfonic acid) (ABTs), Trolox, 2,4,6-Trinitrobenzenesulfonic acid (TNBs), sodium dodecyl sulphate (SDS) and *β*-mercaptoethanol (*β*-ME) were procured from Sigma-Aldrich (USA), Food Pro PNL enzyme was produced by International Flavors and Biosciences (USA).

### 2.3 Removal of non – Collagenous proteins

Pretreatment of starfish specimens with the objective of eliminating non-collagenous proteins was done according the method explained by Vate et al. (2022).The frozen starfish sample was first subjected to a thawing process under cold tap water. Next, the thawed starfish was thoroughly chopped into small pieces 0.5 to 1 cm in size and was subsequently soaked in a NaOH solution of 0.1 M, utilizing a starfish to solution ratio of 1:10 (w/v). The mixture was homogenized (Silverson 5 M, UK) for 2.5 min at 4000 rpm at <4 °C using an ice bath followed by centrifugation at 2000 × g for 2 min. The supernatant obtained was eliminated, and the precipitate, collagen-rich biomass, was blended with chilled water, following adjustment of its pH to 7.4. Thereafter, it was dewatered through centrifugation for 5 mins at 5000 × g[28] and the obtained starfish collagen-rich biomass (SFCB) was used for the pretreatments using US and PEF before the enzymatic hydrolysis. Based on Vate et al. (2022), the ratio of α1 to α2 chains was about 2:1 in all the SF collagen, suggesting that the majority of collagens in SF were type I.

### 2.4 Pretreatment by US and PEF

For US pretreatment, 30 g of SFCB was mixed with 60 ml of miliQ water (50 % w/v) and the dispersion subjected to US processing using a probe sonicator (UIP 1000hdT, Hielscher, Ultrasound Technology, Germany), equipped with a titanium probe with a tip diameter of 22 mm and operating at a frequency of 20 kHz. The following parameters were selected, based on a series of pretrials to find optimum conditions: a power of 750 W was set for the ultrasonic energy, with the amplitude remaining constant. The mixture was treated for a duration of 60 min, with 10 s of active sonication followed by 20 s of idle time, to prevent the temperature from increasing above 20 °C during the sonication process. This resulted in a total US processing time of 20 min. The beaker was placed in an ice bath to avoid the increase in temperature at similar operating conditions, which was continuously monitored by a temperature controller.

**Fig. 1:**
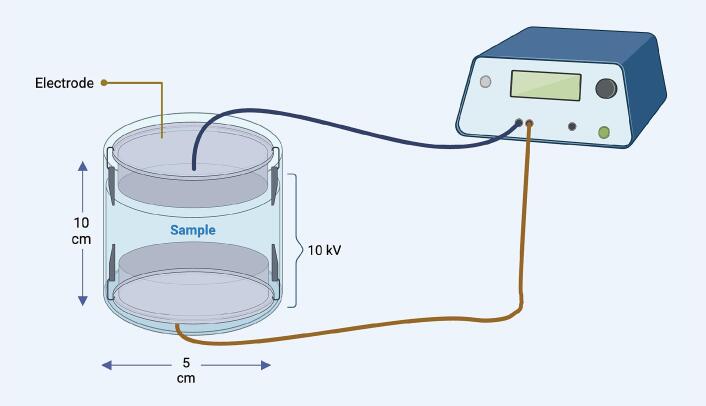
Schematic illustration of the PEF chamber and its geometry.

For PEF pretreatment, 30 g of SFCB was mixed with 60 ml of water (50 % w/v). Before each PEF-treatment, the conductivity of the dispersion was measured using a pH/conductivity meter (Mettler Toledo Seven Go Duo pro, Switzerland) to be specified in the control software of the PEF modulator (0.5 – 0.65 mS/cm). The dispersion was subjected to PEF treatment using a ScandiNova Saligus 10 kW modulator equipped with a cylindrical treatment cell (PG200, ScandiNova, Sweden) at room temperature. The cell had a length of 10 cm (Gap distance between electrodes) and the diameter 5 cm (treatment chamber volume was 204.28 cm^3^), with the applied voltage along its axis of symmetry. A voltage of 10 kV was applied to the dispersion for a duration of 50 s, during which the number of pulses and the width of each pulse were 500 and 10  μs, respectively. The strength of the electrical field was 1 kV/cm. Pulse repetition frequency was 20 Hz. For details, see Fig. 1.

### 2.5 Enzymatic hydrolysis process

After the pretreatments, the temperature of the starfish collagen-rich biomass (SFCB) suspensions was raised to 55 °C, and pH was adjusted to 7.0 using 0.1 M NaOH or HCl as needed. The pH was monitored throughout hydrolysis and adjusted periodically to maintain constant conditions. Food Pro PNL (a neutral protease preparation derived from Bacillus amyloliquefaciens, activity: 1600–1900 AZO/g; International Flavors and Biosciences, USA) was added at two concentrations: 0.5 % and 1 % w/w based on total protein content in the sample, as determined by the Kjeldahl method (conversion factor N × 6.25). Hydrolysis was conducted for 4 h under constant agitation in a temperature-controlled incubator at 55 °C. Aliquots (1 mL) were withdrawn at time intervals of 15, 30, 60, 120, 180, and 240 min for DH analysis. At the end of hydrolysis, the enzyme was inactivated by heating the reaction mixture to 85 °C for 15 min. The hydrolysate was centrifuged (12,000 × g, 20 min), filtered, lyophilized, and stored at −20 °C for subsequent analysis. Hydrolysis under identical conditions, but without US or PEF pretreatment, served as the control.

### 2.6 Measurement of degree of hydrolysis

****The degree of hydrolysis (DH) was determined using the 2,4,6-trinitrobenzenesulfonic acid (TNBS) method as described by Adler-Nissen (1979), with minor modifications. Briefly, 0.5 mL of each hydrolysate sample was mixed with 4 mL of 1 % (w/v) SDS solution. From this, 25 μL aliquots were transferred to test tubes, with 1 % SDS as blank. To each tube, 200 μL of 0.2 M phosphate buffer (pH 8.2) and 200 μL of freshly prepared TNBS reagent were added. Samples were incubated at 50 °C for 60 min in an orbital shaker (150 rpm). The reaction was terminated with 400 μL of 0.1 M HCl, followed by a 30-min incubation. Absorbance was measured at 420 nm using a UV–Vis spectrophotometer (60UV–vis, Agilent technologies, Santa Clara, USA). Leucine was used to prepare a standard curve (0.5–7.5 mM; R^2^ > 0.99). DH was calculated using Eqs. (1), (2):

(1) $$h=\frac{A420-b}{m}$$

(2) $$\mathrm{DH}=\frac{h}{\mathrm{htotal}}\times 100$$

where A_420_ = absorbance at 420 nm, b = Intercept, m= Slope of the calibration curve and h_tot_ = Protein concentrate (that is 8.6 for fish).where h total = 8.6 meq/g protein, based on total peptide bonds in fish proteins (Adler-Nissen, 1979). All measurements were performed in triplicate.

### 2.7 Protein recovery measurement

The total recovery of proteinaceous material using the enzymatic hydrolysis process was estimated based on the Dumas method and calculations of crude protein. The nitrogen content in the lyophilized SFCB hydrolysates and the starfish biomass was measured by a Vario EL cube instrument (Elementar, Langenselbold, Germany), using sulfanilamid as a correction standard and 2 mg samples packed in aluminum foil [29]. Different nitrogen-to-protein conversion factors are generally used for different proteins, and 5.58 is commonly used for collagen. Protein recovery rate was calculated as follows:

(3) $$\mathrm{ProteinRecovery}=\frac{\mathrm{FinalMassofhydrolysate}\times ProteinContentofHydrolysate}{\mathrm{InitialMassofStarfish}\times ProteincontentofStarfish}$$

### 2.8 Antioxidant activity assessment

#### 2.8.1 2,2-Diphenyl-1-picrylhydrazyl (DPPH) radical scavenging activity

DPPH free radical scavenging capacity of the SFCB hydrolysate was measured according to a modified method of Shimada et al. [30]. The reaction substrate which contained different concentrations of the SFCB hydrolysate (2.5, 5, 10, and 15 mg/ml concentrations) was mixed with DPPH solution (0.1 mM in 95 % Methanol) at a ratio of 1:1. The mixtures incubated for 30 min at room temperature and the absorbance was measured at 517 nm using a spectrophotometer (60UV–vis, Agilent technologies, Santa Clara, USA). As blank, distilled water was used instead of the sample. Trolox at concentrations 0.025 to 0.5 mM was used for the standard curve. A lower absorbance indicates higher free radical scavenging properties. Radical scavenging capacity was calculated as follows:

(4) $$\mathrm{DPPH}\%=\frac{\mathrm{BlankAbsorbance}-SampleAbsorbance}{\mathrm{BlankAbsorbance}}\times 100$$

(5) $$IC50\%=\frac{50-b}{m}$$

where b is intercept and m is slope of the calibration curve

#### 2.8.2 2,2′-azino-bis(3-ethylbenzothiazoline-6-sulfonic acid (ABTS) radical scavenging activity

The ABTS scavenging activity of the SFCB hydrolysate was evaluated in accordance to the method described by Obon et al. [31]. In summary, the ABTS solution was prepared by adding 7 mM (36 mg/5 mL) to ABTS (2,2′-azinobis-(3-ethyl-benzothiazoline-6-sulphonic acid)) with 2.45 mM of potassium persulfate (K2S2O8, 4.41 mg/5 mL) in distilled water. To convert the ABTS into its radical cation (ABTS^•+^), the reaction mixture was left in darkness at room temperature for 12–16 h prior to its utilization. The resulting radical solution was diluted with ethanol until it reached an absorbance of 0.7 ± 0.02 at 734 nm. Subsequently, 950 µl of the radical solution was mixed with 50 µl of the SFCB hydrolysate solution, and the absorbance was measured 6 min later at room temperature (734 nm) using a spectrophotometer (60UV–vis, Agilent technologies, Santa Clara, USA).

(6) $$\mathrm{ABTS}\%=\frac{\mathrm{BlankAbsorbance}-SampleAbsorbance}{\mathrm{BlankAbsorbance}}\times 100$$

(7) $$IC50\%=\frac{50-b}{m}$$

where b is intercept and m is slope of the calibration curve.

#### 2.8.3 Reducing power activity assay (RP)

To evaluate the antioxidant activity of peptides, the ability to convert a ferricyanide complex (Fe^3+^) into its reduced form (Fe^2+^) can be used as a measure of reducing power. First, 0.5 mL of each hydrolysate (2.5, 5, 10 mg/ml), 0.5 mL of phosphate buffer (pH 6.6), and 0.5 mL of potassium ferricyanide solution (10 mg/mL) were mixed together. The mixture was then incubated at 50 °C for 20 min. After incubation, 0.5 mL of 10 % trichloroacetic acid was added to the mixture, which was then centrifuged at 5000 × g for 10 min. The clear liquid supernatant was collected and mixed with 1 mL of distilled water and 0.2 mL of ferric chloride solution (0.1 %). This solution was left at room temperature for 10 min, and its absorbance was measured at 700 nm. For the blank sample, the same procedure was followed, but distilled water was used instead of the peptide hydrolysate [32]. An increase in the absorbance indicated higher reducing power [33].

### 2.9 Measuring amino acids profile of the collagen peptides

The amino acid composition of the SFCB hydrolysate was examined using the approach established by Özcan & Şenyuva [34] with certain adjustments. Freeze-dried samples of SFCB hydrolysates (10 mg) were mixed with 4 mL of 6 N HCl and subjected to acid hydrolysis at 110 °C for 24 h under nitrogen to prevent oxidation. The SFCB hydrolysates were diluted using 0.2 M acetic acid and subsequently subjected to automatic injection into an Agilent 1100 HPLC system (Waldbron, Germany) coupled with a mass spectrometer for amino acid separation and quantification. Calibration was performed using a standard mixture of 18 amino acids at known concentrations, allowing for accurate quantification of individual residues in the samples. The analysis was carried out using a reverse-phase C18 column with a binary gradient system consisting of methanol and water with formic and acetic acid additives. Detection was achieved using electrospray ionization in positive mode, with parameters optimized for amino acid identification. The method enabled reliable quantification of most standard amino acids; however, it should be noted that cysteine and tryptophan were not recovered due to their degradation during acid hydrolysis. All measurements were performed in replicate to ensure analytical reproducibility, and results are expressed as micromoles of amino acid per gram of dry hydrolysate.

### 2.10 Analysis of molecular weight distribution of collagen peptides

#### 2.10.1 Size exclusion chromatography (SEC)

The SFCB hydrolysates were dissolved in MilliQ water to a concentration of 10 mg/ml, centrifuged at 10,000 xg for 10  min, and then the resulting supernatant was filtered (0.45 µm) and used for the SEC analysis. For each sample, 20 μl were injected into two sequentially connected Agilent Bio SEC columns (150 Å and 100 Å) maintained at a constant temperature of 25 °C. The mobile phase consisted of 30 % acetonitrile and 0.05 % trifluoroacetic acid in Milli-Q water (v/v). The chromatographic runs were controlled from the Chromeleon software version 7.2 SR 4 (Thermo Schientific, Walham, MA, USA). From the chromatographic runs of both the standards (AdvanceBio SEC 130 Å Protein Standard (Agilent Technologies)) and the SFCB hydrolysates, an UV trace of 214 nm was monitored [35]. The standard was a mixture of proteins with varying molecular weights, including Ovalbumin (45 kDa), Myoglobin (17 kDa), Aprotinin (6.7 kDa), Neurotensin (1.7 kDa), and Angiotensin II (1 kDa).

#### 2.10.2 Sodium dodecyl-sulfate polyacrylamide gel electrophoresis (SDS-PAGE)

SDS-PAGE analysis was conducted in accordance with F. He [36]. The SFCB hydrolysates were dissolved in a 5 % SDS and then mixed with the Laemmli sample buffer (Bio-Rad, USA) at a ratio of 1:2, while also incorporating 10 % β-ME, thereby achieving a final protein concentration of 2 μg protein/μL. Subsequently, 10 μL of each sample, along with 5 μL of an ultra-low range molecular weight marker (1.06–26.6 kDa, sigma, USA), were loaded onto a precasted 16.5 % Mini-protein Tris-Tricine gel (Bio-Rad, USA). The SFCB hydrolysates were then subjected to electrophoresis at a consistent current of 125 V, employing a Mini Protein II unit (Bio-Rad, USA). The gel was fixed in a solution of 5 % glutaraldehyde that had been freshly prepared for a duration of 1 h. Subsequently, the gel underwent a washing process for 5 min, which was repeated three times. Staining was done using 0.02 % (w/v) Coomassie Brilliant Blue R-250 in 10 % (v/v) acetic acid for 1 h followed by destaining 10 % (v/v) acetic acid for 1 h to overnight with several changes of destaining solution. Finally, the gel was imaged using a Bio GelDoc Go Imaging system (Bio-Rad, USA).

### 2.11 Statistical analysis

The pretreatments and hydrolysis of SFCB were conducted at a minimum twice. The determination of significant differences was achieved through the application of analysis of variance (ANOVA) to the data. Utilization of Duncan’s multiple range test enabled the comparison of mean values as outlined by Steel and Torrie (1980), with significance attributed to data when p < 0.05. Statistical analysis was carried out using the Statistical Package for Social Science (IBM SPSS 28.0 for Windows, SPSS Inc., Chicago, IL, USA). Also principal component analysis (PCA) was performed using OriginPro 2023 (OriginLab Corporation, Northampton, MA, USA) to visualize the relationships between antioxidant activity, DH, MW distribution, and AA composition across different treatments.

## 3 Results and discussions

### 3.1 The effect of US and PEF pretreatment on SFCB hydrolysis efficiency (DH)

**Fig. 2:**
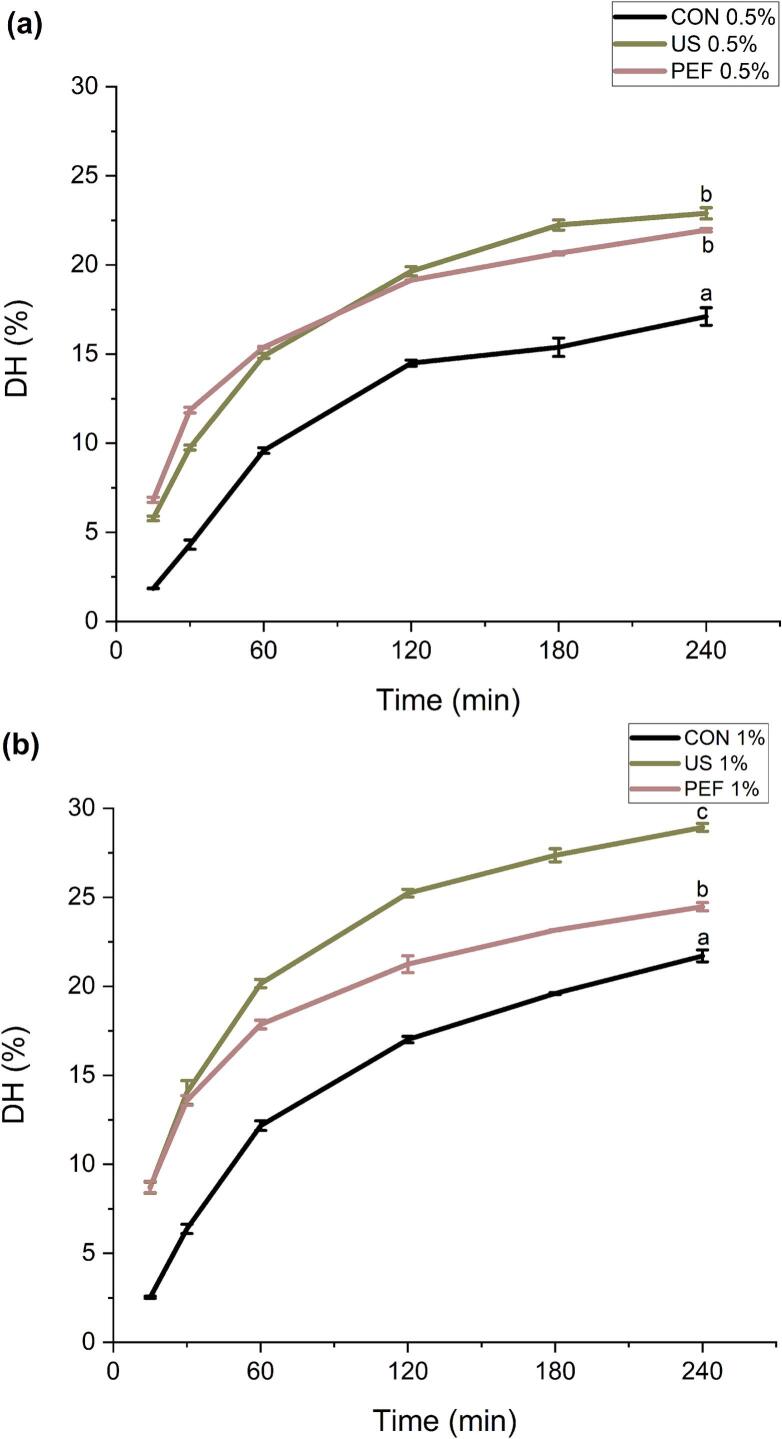
Effect of ultrasonic (US) and pulse electric fields (PEF) pretreatment prior to enzymatic hydrolysis using Food PRO PNL enzyme at 0.5 % (v/w) (a) and 1 % (v/w) (b) on total protein on degree of hydrolysis (DH) of the SFCB. CON; Control (without US and PEF pretreatment). Data are shown as mean value ± SD (n = X).

The quantification of the cleaved peptide bonds is achieved by measuring the DH and it is intimately associated with the functional properties such as solubility, emulsification, foaming, and water- and oil-holding capacities of protein hydrolysates [37]. Regardless of the used enzyme content during the hydrolysis process, the pretreatment with both US and PEF significantly (P < 0.05) increased the DH when compared to the conventional hydrolysis process (Fig. 2 a and b). For instance, after 240 min of hydrolysis at 0.5 % enzyme concentration, DH increased from 17.1 % in the control to 22.9 % with US and 21.9 % with PEF, while at 1 % enzyme concentration, DH rose from 21.7 % in the control to 28.9 % with US and 24.5 % with PEF. In addition, applying the US and PEF pretreatment accelerated the hydrolysis process which resulted in a reduction of the hydrolysis time to reach a specific DH value compared to the conventional hydrolysis process. For example, the DH in the collagen hydrolysate pretreated with US and PEF after 60 min and 120 min reached 20.1 % and 21.2 %, respectively, which were equivalent to the DH achieved using the conventional hydrolysis after 240 min. As expected, the DH increased significantly (P < 0.05) the enzyme to substrate ratio raised from 0.5 to 1 % v/w. However, at a specific time point, the pretreatment with US and PEF compeletly compensated for the lower enzyme concentration, achieving comparable DH even when hydrolysis was conducted with 0.5 % v/w enzyme (Fig. 2a).

These findings suggest that pretreatment with both US and PEF was able to increase susceptibility of SFCB to enzymatic hydrolysis, likely by promoting access of the enzyme to the peptide bonds in the collagen molecules. The cavitation phenomena induced by US can in different ways result in protein conformational changes, unfolding or distruption which can be in favor of enzymatic hydrolysis [38]. US exhibits a distinctive cavitation phenomenon, characterized by the alternation between low and high levels of acoustic pressure that results in the contraction, expansion, or collapse of gas bubbles within the liquid medium. This abrupt collapse event has the potential to generate elevated temperatures, pressures, and a multitude of reactive radicals, consequently leading to the disruption of molecular structures [39]. This disruption encompasses various physical–chemical and mechanical effects, such as the breakdown of hydrogen bonds and van der Waals forces [40], increase in free sulphydryl content and formation of β-sheets and β-turns, as reported for whey proteins pretreated with US [41]. Our findings are in agreement with recent literature showing that physical pretreatments such as US and PEF can enhance the degree of hydrolysis by disrupting protein structures and increasing enzyme accessibility, thereby facilitating the release of antioxidant peptides Habinshuti et al. [42].

The increase in DH following PEF pretreatment can be attributed to its ability to inducing protein unfolding using electric field-Induced polarization. Proteins are polar molecules, and exposure to high-voltage electric fields (typically 1–50 kV/cm) causes dipole alignment and redistribution of charges [43]. These structural modifications enhance the susceptibility of collagen to enzymatic attack. Our results aligns with previous studies in which PEF pretreatment amplified the DH of Antler residue where a raise in electric field intensity from 5 kV/cm to 20 kV/cm, yielded a substantial increase in DH from 2.1 % to 17.3 % [44]. Mikhaylin et al. [22] also showed that, under optimal conditions, the DH of β-lactoglobulin increased by 80 % as results of PEF pretreatment. This enhancement was attributed to the ability of high voltage electric field to expose active sites within the protein molecule for the nucleophilic enzymatic action. It has been previously reported that protein unfolding induced by other non-thermal processing methods, notably high-pressure processing (HPP), enhances enzyme accessibility and promote hydrolysis in dairy protein systems [45]. Although HPP operates through hydrostatic pressure, and PEF and US use electric fields and cavitation effects respectively, the outcome enhanced hydrolysis efficiency through structural modification appears consistent across these technologies. Our findings thus support the broader concept that non-thermal pretreatments, regardless of their precise mechanism, can improve enzymatic processing by altering protein conformation and facilitating enzymatic access to cleavage sites a good replaced for the classic thermal pretreatments.

When comparing the effect of the two pretreatment methods, at 0.5 % v/w enzyme to substrate ratio, there was no significant difference between DH obtained with the pretreament via US and PEF. However, by increasing the enzyme ratio to 1 %, v/w, the US pretreatment resulted in significantly (P < 0.05) higher DH (around 5 %) than the PEF pretreatment. The process, involving US and employment of 1 % enzyme, exhibited the highiest DH (27 %) after 240 min. This implies that the US pretreatment was more effective than PEF to promote the susceptibility of SFCB to enzymatic hydrolysis, but the sites exposed for the hydrolysis have been only accessible at an excessive amount of enzyme. There are no other reports comparing US and PEF as pretreatment for enzymatic hydrolysis of collagen but Uluko et al. [46] reported that the US was the most effective pretreatment compared with thermal and microwave pretreatments for producing antioxidant peptides from milk protein concentrate using enzymatic hydrolysis. Consistent with our findings, a study reported that probe-type US pretreatment significantly enhanced DH (69.90 %) compared to US bath (62.28 %) and control (43.89 %), indicating the role of US in improving collagen peptide generation and functional properties [47].

### 3.2 The effect of US and PEF pretreatment on (hydrolysis efficiency) recovery of SFCB hydrolysate

**Fig. 3:**
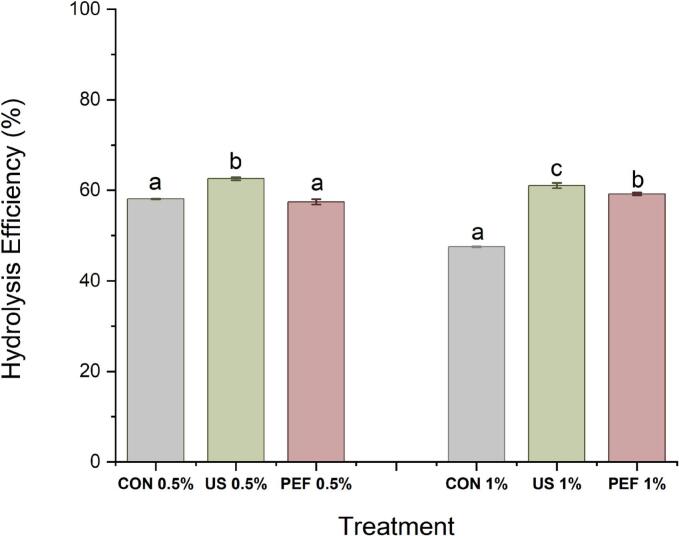
Effect of ultrasonic (US) and pulsed electric field (PEF) pretreatment followed by enzymatic hydrolysis on the recovery of SFCB hydrolysate from starfish with two concentrations of enzyme; 0.5 % and 1 % v/w of total protein. CON0.5 %: Conventional hydrolysis with 0.5 % (v/w) enzyme, US0.5 %: ultrasound pretreatment followed with hydrolysis with 0.5 % enzyme (v/w), PEF0.5 %: pulsed electric field pretreatment followed with hydrolysis with 0.5 % enzyme (v/w). CON 1 %: Conventional hydrolysis with 1 % (v/w) enzyme, US 1 % (v/w): ultrasound pretreatment followed with hydrolysis with 1 % enzyme, PEF 1 %: pulsed electric field pretreatment followed with hydrolysis with 1 % enzyme. The same letter indicates no statistically significant difference between groups and different letters indicate statistically significant differences between groups at the p < 0.05 level.

**Fig. 4:**
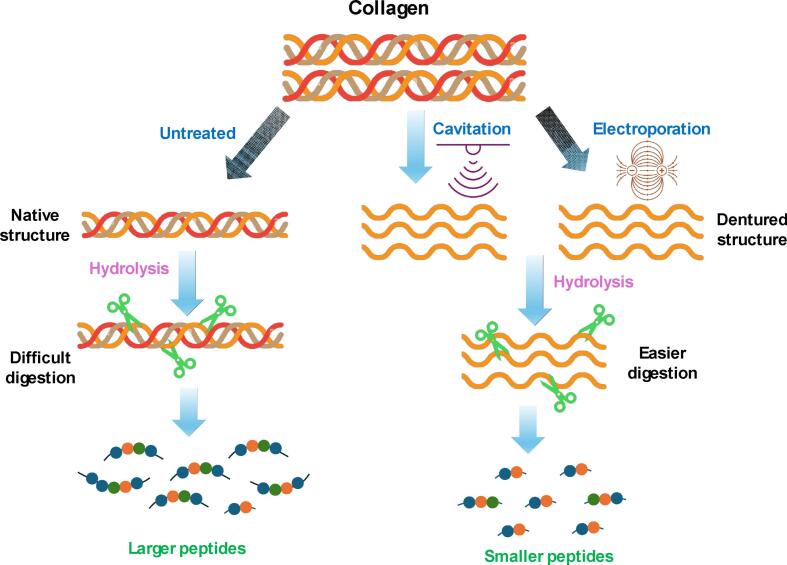
Schematic illustration of methods for enhancing collagen susceptibility to enzymatic hydrolysis: untreated collagen vs. effects of cavitation (US) and electroporation (PEF).

Fig. 3 shows the impacts of the US and PEF pretreatment on the collagen hydrolysate recovery (hydrolysis efficiency) in comparison to the conventional enzymatic hydrolysis process. The recovery for SFCB treated with US increased significantly (P < 0.05) and reached approximately 62.5 and 61.0 % w/w (using both 0.5 % and 1 % v/w enzyme concentrations), representing the highiest recovery. Particularly at an enzyme concentration of 1 %, significant (P < 0.05) differences in protein hydrolysate retrieval were noted between the groups subjected to pretreatment (US and PEF) and the control group. The protein recovery percentages obtained were 61.0 % for US, 59.1 % for PEF, and 47.5 % for the control group (Fig. 4).

Other researchers have demonstrated that US has the potential to enhance the efficiency of enzymatic hydrolysis for example using cellulase where determination of kinetic parameters as well as observation of protein structural changes were useful for elucidating the action mechanism [48]. M. Wang et al. [49] noted that the application of PEF improved protein extraction efficiency from both rainbow trout and sole skins compared to untreated controls, indicating that PEF enhances cell disruption and promotes the release of protein-bound compounds. In a study by L. He et al. [50], it was demonstrated that the recovery of protein hydrolysates significantly increased following ultrasonication at 200–300 W compared to the control group. This enhancement was attributed to the ability of US to disrupt the collagen structure, unfold the triple-helix configuration, and increase molecular mobility, thereby improving protein solubility and enabling more efficient enzymatic hydrolysis and peptide release.

### 3.3 The effect of US and PEF pretreatment on molecular weight distribution of peptides

**Fig. 5:**
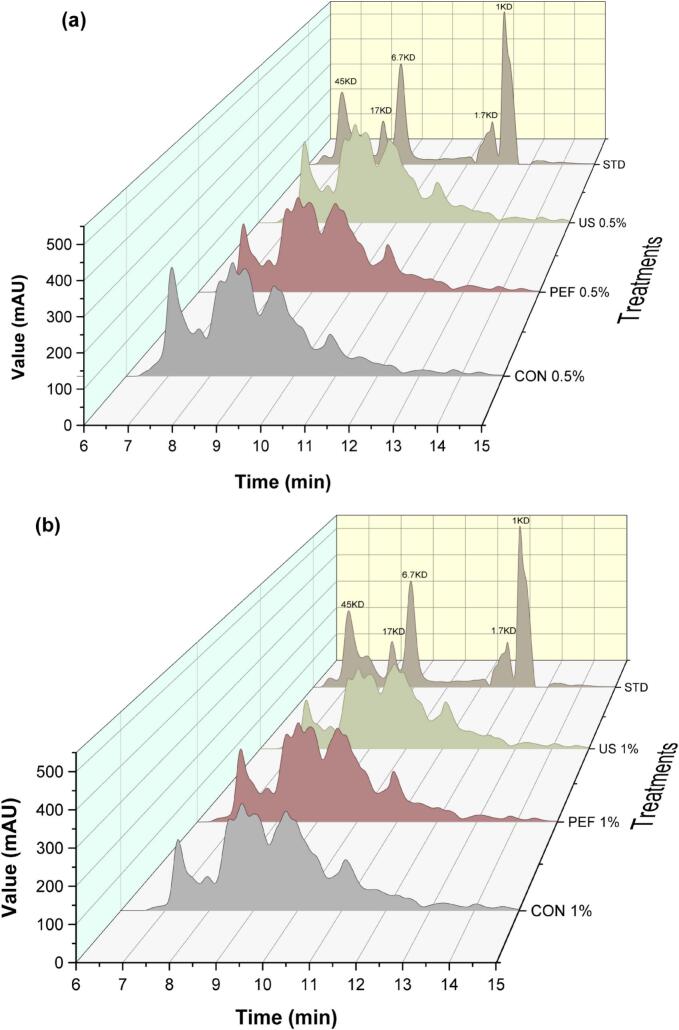

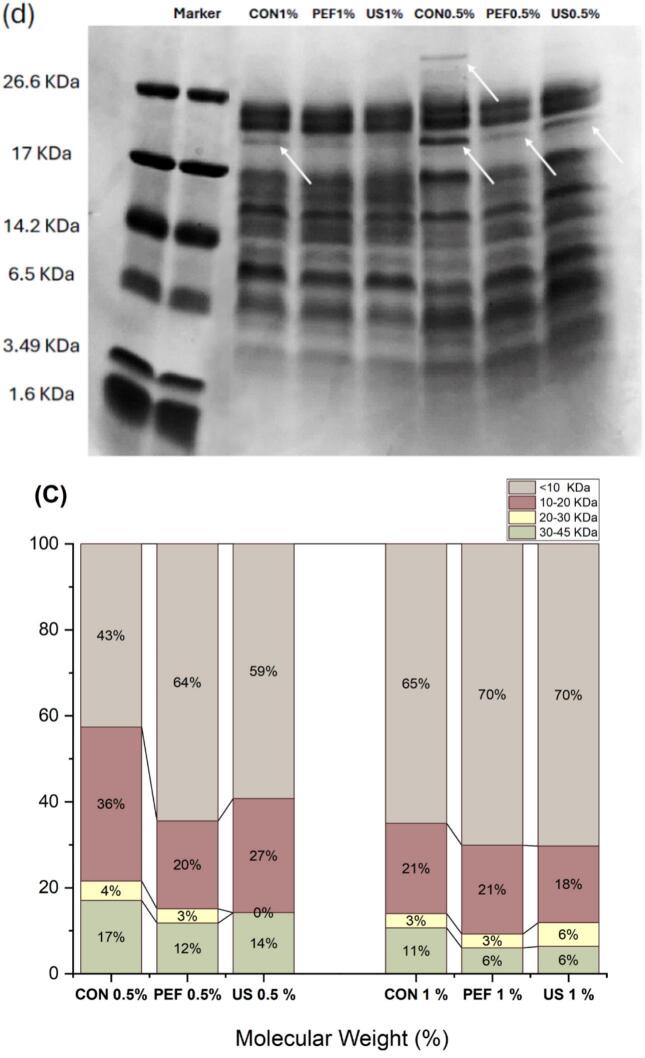
Size exclusion chromatograms showing the effect of ultrasonic (US) and pulse electric fields (PEF) pretreatment during enzymatic hydrolysis (a) with 0.5 % v/w and (b) 1 % v/w enzyme on molecular weight of the SFCB hydrolysates, (c) their relative percentage of total peak area and (d) their SDS-PAGE. CON 0.5 %: Conventional hydrolysis with 0.5 % enzyme, US0.5 %: ultrasound pretreatment followed with hydrolysis with 0.5 % enzyme, PEF0.5 %: pulsed electric field pretreatment followed with hydrolysis with 0.5 % enzyme. CON 1 %: Conventional hydrolysis with 1 % enzyme, US 1 %: ultrasound pretreatment followed with hydrolysis with 1 % enzyme, PEF 1 %: pulsed electric field pretreatment followed with hydrolysis with 1 % enzyme. In the SDS-PAGE gel, 10 μl of samples were loaded to each lane.

The distribution of SFCB hydrolysates was categorized into four intervals based on their MW, which were <10 kDa, 10–20 kDa, 20–30 kDa, and 30–45 kDa, as shown in Fig. 5, Fig. 5, Fig. 5. The proportion of SFCB hydrolysate <30 kDa, and especially <10 kDa, significantly (P < 0.05) increased in the hydrolysates produced with the aid of the US and PEF pretreatment, compared with the those produced by the conventional hydrolysis (control). Applying PEF and US pretreatment at an enzyme concentration of 0.5 % v/w increased the proportion of collagen hydrolysates with a MW < 10KDa to 64 % and 59 %, respectively, compared with to only 43 % with the conventional method.

The percentage of LMW peptides exhibited a substantial increase by increasing the quantity of the enzyme to 1 % v/w in all the samples. The MW distribution of the hydrolysates obtained using the different methods, as visualized via SDS-PAGE (Fig. 5d), supported the findings seen with SEC. In the sample produced using the conventional hydrolysis at 0.5 % v/w enzme, there was a number of bands with MW > 26.6 KDa that were absent in the US- and PEF-treated samples. SDS-PAGE also supported the finding that the hydrolysis process was more efficient by increasing the enzyme content to 1 %v/w. Based on the results obtained from SEC analysis, further supported by SDS-PAGE, it can be inferred that approximately 94 % of the produced SFCB hydrolysates exhibited a MW < 30 kDa when subjected to both US and PEF pretreatments followed by hydrolysis with 1 % v/w enzyme. However, in the absence of any pretreatment, the proportion of the hydrolysates with a MW < 30 kDa was only approximately 89 %. Similarly, Hao et al. [51] reported that the content of LMW peptides in enzymatically produced porcine bone collagen hydrolysate increased by applying an US treatment.

The ratio of LMW peptides in the collagen hydrolysates was consistent with the DH results (see Fig. 2). As initially hypothesized, this correlation may be attributed to the ability of appropriately applied US treatment to induce protein unfolding via the cavitation effect [52]. The resulting increase in the contact area between the enzyme and the substrate—i.e., collagen molecules—likely contributed to the higher DH and increased production of LMW peptides. This effect was more pronounced at the lower enzyme-to-substrate ratio, likely due to the greater need to enhance enzyme access to peptide bonds under such conditions. Xu et al. [18] reported that collagen treated with US for 10 min caused a disappearance of β-chains and γ-chains, and the color of the α-chains became lighter as the US time increased. This phenomenon likely increased susciptability of collagen for the enzymatic hydrolysis.

When it comes to PEF, previous research has demonstrated that protein molecules exhibit polarization at low intensity of PEF, leading to gradually increased exposure of their hydrophobic amino acids to the surrounding solvent as the electric field intensity increases. As a consequence, the unfolded proteins may aggregate through the formation of weak covalent and non-covalent bonds [53]. Once the PEF intensity surpasses a certain threshold, the thermal effect induced by the electric arc becomes significant, causing denaturation and aggregation of heat-sensitive proteins [21]. Additionally, investigations have revealed that PEF has the capability to disrupt the secondary structure of proteins, resulting in an increase in the proportion of β-sheets and a decrease in the content of α-helices [54,55]. In the present study, the applied electrical field may have denatured the collagen triple helical structure, breaking down its inter- and intramolecular crosslinks, facilitating the access of the enzyme to the peptide bond, especially at lower enzyme content. This may thus explain the very positive effect of PEF pretreatment on the generation of LMW peptides especially at 0.5 % v/w enzyme.

The reduction in molecular weight (MW) of collagen hydrolysates induced by US and PEF pretreatments may positively influence their bioactivity. These changes in MW distribution, as previously reported, play a more critical role in bioactivity than the overall degree of hydrolysis (DH). For instance, B. Wang et al. [56] found no direct correlation between DH and radical scavenging capacity, emphasizing instead that low molecular weight (LMW) peptides are primarily responsible for antioxidant activity. Antioxidative peptides are often composed of fewer than 20 amino acid residues and are enriched in hydrophobic amino acids such as proline, histidine, and tyrosine, which contribute to their radical scavenging potential [57]. Moreover, the small size and hydrophobicity of these peptides enhance their ability to cross the intestinal barrier via passive transport, increasing their likelihood of exerting systemic biological effects [58]. Thus, the ability of US and PEF pretreatments to promote the formation of LMW peptides may be a key factor in enhancing the functional properties of collagen-derived hydrolysates.

### 3.4 The effect of US and PEF pretreatment on amino acid composition of peptides

Beyond size, the antioxidant characteristics of peptides are intricately linked to their amino acid composition, the sequence of these amino acids and their hydrophobic properties [59]. All the produced hydrolysates exhibited a relatively similar amino acid composition to that of the parent starfish collagen, previously reported by Vate et al. [28], which was notably abundant in glycine, proline, alanine, aspartic acid, glutamic acid and hydroxyproline. As shown in Table 1, both US and PEF pretreatments followed by enzymatic hydrolysis led to a noticeable elevation in the overall quantity of basic amino acids, including lysine, arginine, and histidine in the produced SFCB hydrolysates compared with the conventional enzymatic hydrolysis. These findings align with the observations by Zou et al. (2016a) who noted that ultrasonicated porcine cerebral hydrolysate exhibited a notable abundance of basic amino acids. It was previously reported that the elevated levels of glycine and proline contributed to the augmented antioxidant activity observed in fish skin gelatin compared to its meat protein [60]. It is pertinent to highlight that basic amino acids such as lysine, arginine, and histidine possess the ability to serve as hydrogen donors and exhibit substantial efficacy in scavenging free radicals (Zou et al., 2016b). Aromatic amino acids such as phenylalanine, tyrosine, and tryptophan are also regarded as efficient radical-scavenging agents, due to their phenolic groups [61]. The cumulative quantities of aspartic acid and glutamic acid present in the SFCB hydrolysates produced with US pretreatment using 0.5 % and 1 % enzyme were 184.13 and 168.35 mg/g hydrolysate, respectively, which was significantly higher than in the control group (171.27 and 161.57 mg/g hydrolysate, respectively). Furthermore, J. Liu et al. [61] found that the antioxidative potential of bovine collagen peptides was associated with the levels of C-terminal amino acids (arginine and tyrosine) as well as N-terminal amino acids (histidine, phenylalanine, and leucine).

**Table 1:** Effect of ultrasonic (US) and pulsed electric fields (PEF) pretreatment on amino acid profile of SFCB hydrolysates (mg/g hydrolysate). CON0.5%: Conventional hydrolysis with 0.5% enzyme, US0.5%: ultrasound pretreatment followed with hydrolysis with 0.5% enzyme, PEF0.5%: pulsed electric field pretreatment followed with hydrolysis with 0.5% enzyme. CON 1%: Conventional hydrolysis with 1% enzyme, US 1%: ultrasound pretreatment followed with hydrolysis with 1% enzyme, PEF 1%: pulsed electric field pretreatment followed with hydrolysis with 1% enzyme.

	CON 0.5	US 0.5	PEF 0.5	CON 1	US 1	PEF 1
LYS	22.83 ± 0.22^b^	22.20 ± 0.75^ab^	22.97 ± 0.08^b^	22.92 ± 1.04^b^	22.93 ± 0.71^b^	21.24 ± 0.16^a^
ARG	75.65 ± 2.9^b^	79.21 ± 0.51^b^	73.26 ± 0.36^b^	63.99 ± 3.08^a^	73.61 ± 4.5^b^	73.00 ± 2.2^b^
HIS	11.55 ± 0.22^a^	11.68 ± 0.34^a^	11.18 ± 0.14^a^	10.93 ± 0.59^a^	11.48 ± 0.28^a^	11.07 ± 0.1^a^
GLY	169.23 ± 7.8^ab^	187.80 ± 3.8^b^	175.56 ± 6.7^b^	152.48 ± 9.7^a^	179.07 ± 10^b^	169.06 ± 5.4^a^
SER	66.62 ± 0.12^ab^	69.48 ± 1.4^b^	65.30 ± 2^ab^	62.34 ± 3.4^a^	65.99 ± 1.1^ab^	65.48 ± 1.6^ab^
ALA	67.14 ± 0.85^ab^	73.83 ± 1.8^c^	70.51 ± 2.1^bc^	62.28 ± 4.4^a^	69.71 ± 2.4^bc^	65.03 ± 1.4^ab^
THR	29.77 ± 0.51^a^	30.39 ± 0.59^a^	30.40 ± 0.01^a^	29.27 ± 1.2^a^	30.25 ± 0.65^a^	29.60 ± 0.07^a^
ASP	71.61 ± 4.7^ab^	77.04 ± 1.7^b^	70.60 ± 1.01^a^	66.70 ± 0.58^a^	71.17 ± 2.3^ab^	66.74 ± 0.12^a^
GLU	99.66 ± 2.6^b^	107.09 ± 2.1^c^	98.75 ± 3.02^ab^	94.88 ± 1.2^ab^	97.18 ± 0.99^ab^	94.27 ± 1.2^a^
PRO	81.73 ± 2.6^ab^	83.29 ± 3.7^b^	82.46 ± 1.1^ab^	77.96 ± 0.69^ab^	78.58 ± 0.46^ab^	77.44 ± 1.3^a^
VAL	24.90 ± 0.01^b^	26.86 ± 0.65^c^	26.21 ± 0.06^c^	22.34 ± 0.15^a^	26.39 ± 0.54^c^	24.66 ± 0.01^b^
MET	21.21 ± 0.5^c^	18.83 ± 0.65^a^	19.74 ± 0.13^ab^	19.61 ± 0.03^ab^	19.49 ± 0.59^ab^	20.51 ± 0.27^bc^
TYR	19.90 ± 0.43^ab^	20.56 ± 0.94^b^	19.11 ± 0.04^a^	20.07 ± 0.08^ab^	19.23 ± 0.09^a^	19.55 ± 0.42^ab^
LEU	26.33 ± 1.2^ab^	27.07 ± 0.54^ab^	26.82 ± 0.26^ab^	26.00 ± 0.34^ab^	27.42 ± 0.69^b^	25.47 ± 0.66^a^
ILE	25.80 ± 0.53^abc^	27.26 ± 0.55^d^	26.30 ± 0.5^bcd^	24.75 ± 0.47^a^	26.96 ± 0.78^cd^	25.05 ± 0.12^ab^
PHE	12.77 ± 0.1^a^	13.14 ± 0.19^a^	12.79 ± 0.01^a^	12.45 ± 0.86^a^	13.13 ± 0.5^a^	12.29 ± 0.26^a^
HYD-PRO	25.26 ± 0.39^bc^	24.84 ± 0.4^ab^	24.09 ± 0.29^a^	25.40 ± 0.58^bc^	26.15 ± 0.07^c^	25.11 ± 0.7^ab^
HyPho-AA	289,65	300,67	295,23	274,66	291,93	280,05
Total AA	851.98	900.57	856.05	794.37	858.73	825.57

Hydrophobic amino acids (HyPho-AA).

Different letters in each row indicate statistically significant differences between groups at the p < 0.05 level.

In comparison to the control group, the SFCB hydrolysates produced with US and PEF pretreatment exhibited significantly highier overall concentrations of C-terminal and N-terminal amino acids, measuring approximately 145, 141, and 133 mg/g for US, PEF, and control hydrolysates, respectively. In addition, a significant increase in the total quantity of hydrophobic amino acids (HyPho-AA) such as leucine, isoleucine, valine, methionine, phenylalanine, alanine, and proline by US and PEF pretreatment was observed. It has been reported that the presence of a large amount of hydrophobic amino acids (HyPho-AA) can effectively enhance the solubility of peptides in lipid phases and facilitate their reaction with lipophilic free radicals [62,63].

Overall, US and PEF pretreatments before enzymatic hydrolysis significantly influenced the amino acid composition of the resulting collagen peptides. These changes can be attributed to structural modifications in the collagen matrix induced by the pretreatments, which enhance enzyme accessibility and alter cleavage patterns. Both US and PEF treatments increased the release of key HyPho-AA such as glycine, alanine, valine, and proline, particularly at 0.5 % enzyme concentration. Arginine content also increased notably in US- and PEF-treated samples, especially at 1 % enzyme, suggesting improved release of basic amino acids with known antioxidant and metal-chelating properties. The total HyPho-AA increased in the US- and PEF-treated groups compared to the control, and the total amino acid yield followed a similar trend, indicating more extensive hydrolysis. The relevance of amino acid profiling and its association with bioactivity has been demonstrated by Akbarbaglu et al. [62], who fractionated LMW peptides (<3 kDa) from apricot kernel protein hydrolysates confirmed that targeted release of specific peptides enriched in hydrophobic, aromatic, and positively charged amino acids correlated with enhanced antioxidant, antibacterial, and ACE-inhibitory activities.

### 3.5 The effect of US and PEF pretreatment on antioxidant activity of collagen hydrolysate

**Fig. 6:**
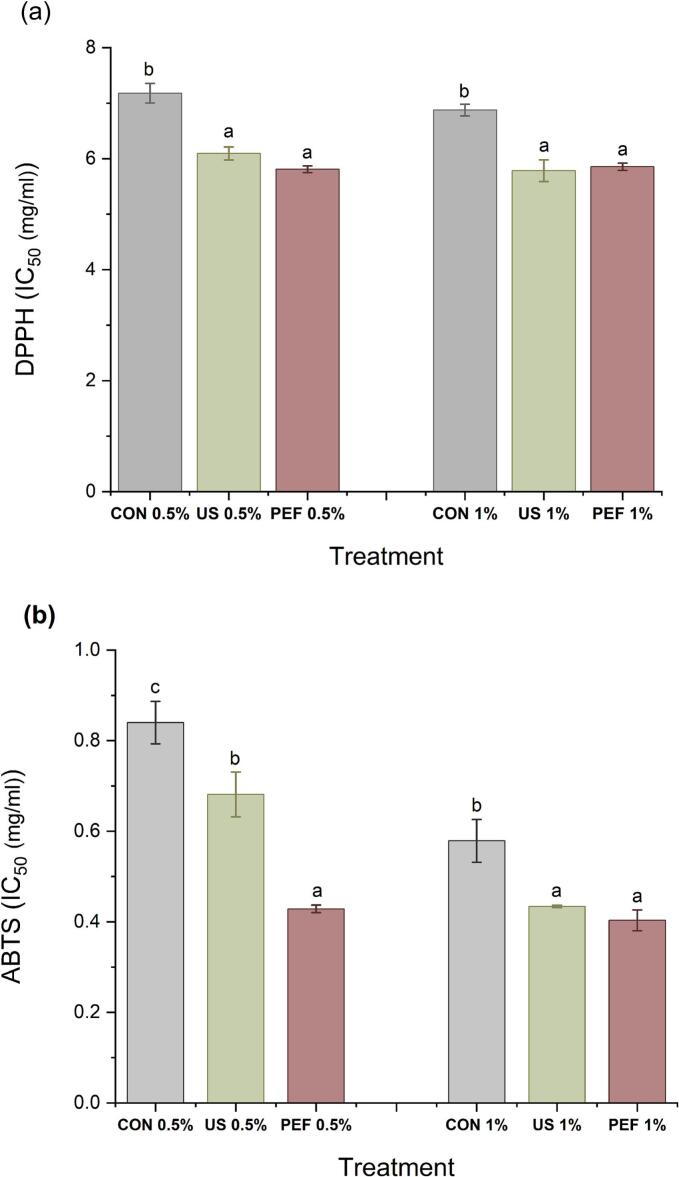

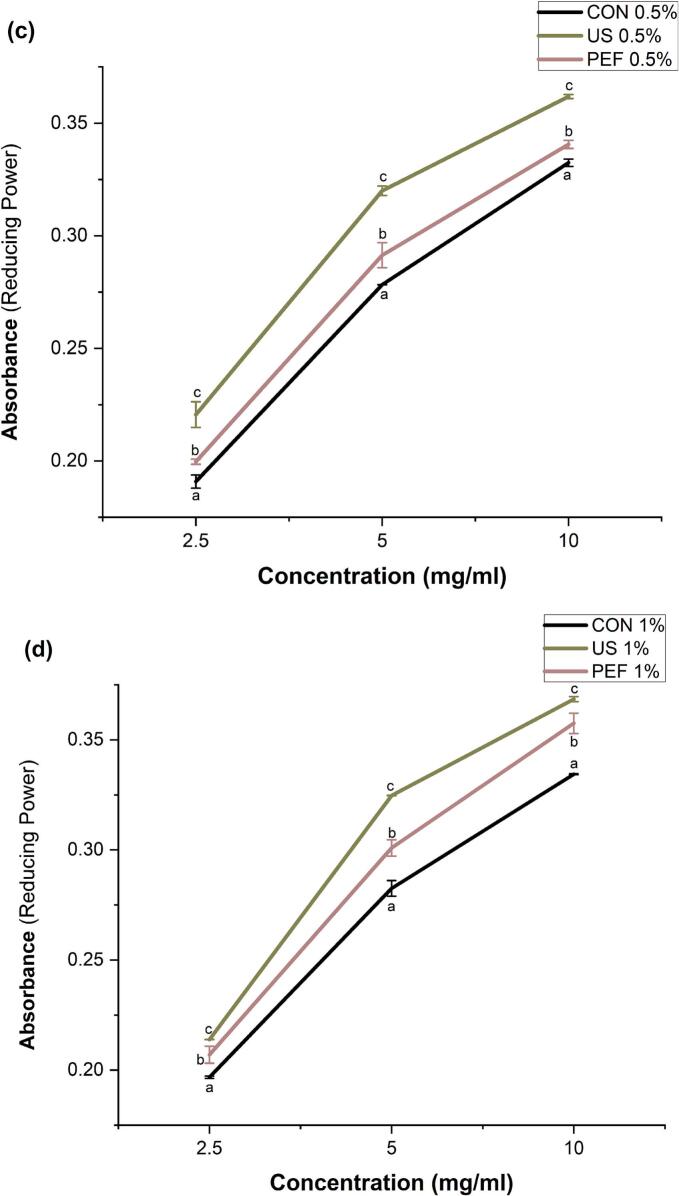
Effect of ultrasonic (US) and pulsed electric fields (PEF) pretreatment on antioxidant activity (ABTS, DPHH radical scavenging and reducing power) of SFCB hydrolysates. CON0.5 %: Conventional hydrolysis with 0.5 % enzyme, US0.5 %: ultrasound pretreatment followed with hydrolysis with 0.5 % enzyme, PEF0.5 %: pulsed electric field pretreatment followed with hydrolysis with 0.5 % enzyme. CON 1 %: Conventional hydrolysis with 1 % enzyme, US 1 %: ultrasound pretreatment followed with hydrolysis with 1 % enzyme, PEF 1 %: pulsed electric field pretreatment followed with hydrolysis with 1 % enzyme. The same letter indicates no statistically significant difference between groups and different letters indicate statistically significant differences between groups at the p < 0.05 level.

The DPPH and ABTS assays were employed to evaluate the radical scavenging capacity of SFCB hydrolysates, while reducing power was assessed via the ferricyanide complex reduction assay to cover various mechanisms of antioxidant activity. Radical quenching serves as a fundamental mechanism employed by antioxidants for the purpose of impeding oxidative processes. 2,2-diphenyl-1-picrylhydrazyl (DPPH), possessing a solitary unpaired electron, can efficiently absorb hydrogen from free radical scavengers, enhancing its stability [64]. Pretreatment with both PEF and US, in combination with enzymatic hydrolysis, significantly (p < 0.05) enhanced ABTS and DPPH radical scavenging activity and reducing power compared to the conventional enzymatic hydrolysis (Fig. 6). At an enzyme concentration of 0.5 %, the IC50 values for ABTS radical inhibition were 0.68, 0.43, and 0.84 mg/mL for hydrolysates produced with US pretreatment, PEF pretreatment, and control, respectively. These results align with the higher proportion of low molecular weight peptides (<10 kDa) observed in the hydrolysates produced with US and PEF pretreatments compared to the conventional method. Notably, at the lower enzyme concentration (0.5 % w/w), PEF pretreatment yielded hydrolysates with superior ABTS radical scavenging activity compared to US pretreatment, correlating with the greater proportion of peptides <10 kDa in the PEF-treated samples (Fig. 5a-c). These LMW peptides are known to possess superior radical scavenging capacity due to their higher diffusibility, better accessibility to reactive sites, and stronger ability to donate electrons or hydrogen atoms. In particular, their small size allows for more efficient interaction with free radicals in DPPH and ABTS assays, leading to enhanced scavenging activity [65]. These findings are consistent with the broader role of PEF in enhancing peptide bioactivity, as reported by Tadesse & Emire [66] who highlighted that PEF treatment facilitates conformational loosening and microstructural disruption of protein matrices, thereby improving enzymatic access and accelerating the release of antioxidant peptides. In line with this, the superior ABTS scavenging and reducing power observed in PEF-pretreated hydrolysates, especially at lower enzyme concentrations, can be attributed to the increased liberation of LMW peptides with greater accessibility and reactive amino acid exposure. Such effects reinforce the utility of PEF as a scalable, non-thermal pretreatment method in functional food applications aimed at enhancing peptide derived antioxidant functionality. Moreover, shorter peptides often have exposed amino acid residues, especially hydrophobic, aromatic, and basic residues, that can directly participate in redox reactions, contributing to increased reducing power [66,67]. Therefore, the shift toward lower molecular weight peptide profiles observed in the US- and PEF-pretreated hydrolysates is likely one of the key contributor to the enhanced antioxidant performance measured across both assays.

As can be seen in Fig. 6a, the DPPH radical scavenging activity of the hydrolysates in control was independent of the used enzyme concentration. However, at 1 % enzyme, the inhibition of the DPPH radicals exhibited the same pattern as 0.5 % enzyme, with a greater value in samples that underwent pretreatment with PEF and US. Similarly, Xu et al. [18] reported that a collagen hydrolysates derived from deer tendon, assisted with different US pretreatment times, exhibited a noteworthy decline in the IC50 value for DPPH scavenging. The found that as the duration of US increased to 60 min, the IC50 value of their collagen hydrolysate decreased by 8.74 ± 0.76 % compared to the control. Increasing the enzyme concentration to 1 % (w/w) enhanced ABTS inhibition across all sample types, with both PEF and US pretreatments continuing to provide significantly higher antioxidant activity than the controls (see Fig. 6b). These findings also align with the significant redulction in the proportion of LM peptides (<10 kDa) with increasing the enzyme to 1 % and its combination with US and PEF. These findings are in agreement with Kangsanant et al. [68] who reported that US pretreatment and ultrasonic-assisted enzymatic hydrolysis of tilapia protein significantly enhanced antioxidant properties, including DPPH radical scavenging and ferric reducing antioxidant power. Their study demonstrated that US pretreatment altered the structural conformation of proteins, increasing substrate accessibility to enzymatic cleavage and promoting the release of LMW peptides with enhanced bioactivity. This supports our observation that increasing enzyme concentration to 1 % in combination with US or PEF pretreatments led to a notable reduction in the proportion of large peptides and a corresponding improvement in radical scavenging activity. These structural modifications, facilitated by physical pretreatments, play a pivotal role in enhancing functional properties of marine collagen hydrolysates. These results are further supported by Indriani et al. [69], who reported that ultrasound-assisted enzymatic hydrolysis of collagen from Asian bullfrog skin significantly improved antioxidant activities, including DPPH and ABTS radical scavenging, compared to the ontrol. The improvement was attributed to the ability of ultrasound to disrupt the triple-helix structure of collagen, facilitating greater enzyme access and enhancing the release of short-chain peptides with high antioxidant potential. Similarly, in our study, US pretreatment promoted the generation of LMW peptides and improved radical scavenging activity, reinforcing the role of US as a structural modifier that enhances hydrolysis efficiency and bioactive peptide release. These results also align with those of Akbarbaglu et al. [62], who demonstrated that enzymatic hydrolysis of apricot kernel protein, accompanied by increased DH from approximately 3 % to 38 %, significantly enhanced antioxidant activities, with DPPH and ABTS radical scavenging capacities reaching nearly 83 % and 88 %, respectively. The improved radical scavenging activity of the collagen hydrolysates following US and PEF pretreatments can be closely linked to their enhanced amino acid profiles too. As shown in our results, US and PEF pretreamtnets increased presence of hydrophobic, and basic amino acids such as leucine, phenylalanine, lysine, and histidine which likely contributed to stronger radical scavenging through electron donation and hydrogen transfer [61,70]. Similar observations were reported by Akbarbaglu et al. [62], emphasizing that both peptide size and composition, influenced by targeted pretreatments, play a critical role in determining antioxidant potential. Our findings further align with Akbarmehr et al. [71], who reported that hydrolysates generated with pepsin, characterized by higher DH and favorable amino acid profiles, exhibited superior antioxidant activity.

Both US and PEF pretreatments also significantly (p < 0.05) enhanced the reducing power of the SFCB hydrolysates, with US showing the greatest improvement. This can be also attributed to the formation of LMW peptides enriched in HyPho-AA as shown in Fig. 5 and Table 1. These amino acids, including leucine, phenylalanine, tyrosine, and tryptophan, possess electron-donating capabilities, enhancing radical scavenging and reducing activity [62,71,72]. Our amino acid analysis confirmed that US and PEF pretreatments increased the levels of hydrophobic amino acids to 300.67 mg/g and 295.23 mg/g, respectively, compared to 289.65 mg/g in the control (see Table 1).

In summary, US and PEF pretreatments significantly enhanced the antioxidant activity of SFCB hydrolysates by increasing DH, promoting the release of low molecular weight peptides, and enriching the hydrolysate in radical-scavenging amino acids.

### 3.6 Comparative assessment of PEF, US and control with principal component analysis

**Fig. 7:**
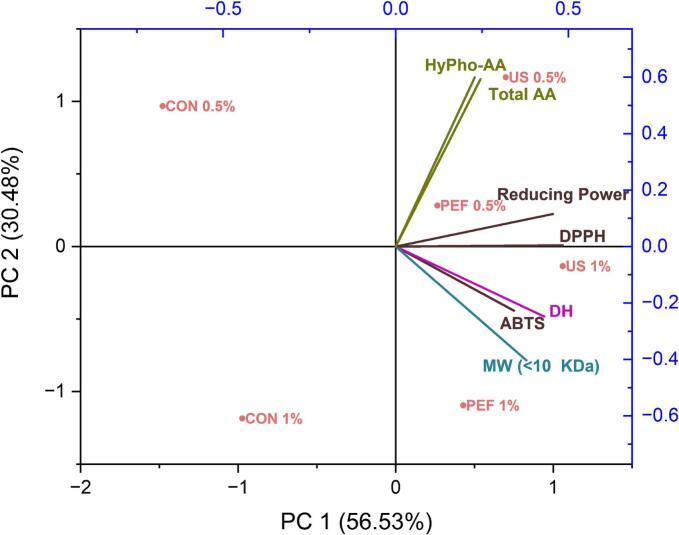
PCA biplot showing the relationship between antioxidant activity, DH, MW (<10 kDa), AA composition, and SFCB hydrolysates produced by US, PEF, and control treatments.

The principal component analysis (PCA) was used to gain a comprehensive overview of the relationship between AA composition, DH, MW distribution, and antioxidant activity in the SFCB hydrolysates subjected to PEF, US, and conventional enzymatic hydrolysis (Fig. 7). The first two principal components (PC1 and PC2) together explained 86.01 % of the total variance, with PC1 accounting for 56.53 % and PC2 for 30.48 %.

The PCA biplot clearly differentiated the hydrolysate samples based on their treatment and enzyme concentrations. Notably, both PEF and US treatments clustered distinctly from the control groups, indicating their substantial influence on the structural and functional properties of the hydrolysates. US pretreatment at 0.5 % enzyme concentration showed a strong positive correlation with hydrophobic amino acids (HyPho-AA) and total amino acid levels, aligning with prior reports that ultrasonic cavitation disrupts protein aggregates, improves enzymatic accessibility, and enhances the yield of bioactive, hydrophobic peptide sequences [50,70]. These structural modifications are known to boost radical scavenging potential through increased availability of electron-donating residues such as leucine, phenylalanine, and tyrosine [5].

Interestingly, hydrolysates produced with US and PEF pretreatments at 1 % enzyme concentration cluster in proximity to variables such as DH, ABTS radical scavenging activity, and the proportion of low molecular weight peptides (<10 kDa). This reflects the enhanced hydrolysis efficiency and improved antioxidant activity at higher enzyme loading, supported by literature demonstrating the synergistic effects of pretreatment and enzymatic hydrolysis on peptide functionality [18,62]. In contrast, the control hydrolysates produced without pretreatments cluster separately along negative PC1 and PC2 axes, particularly at higher enzyme concentrations (1 % w/w). This separation reflects their comparatively lower levels of DH, antioxidant capacity, and favorable amino acid composition, underscoring the limited effectiveness of conventional enzymatic hydrolysis alone. Collectively, these PCA findings confirm that both PEF and US pretreatments significantly improved the structural and functional attributes of marine collagen hydrolysates relative to control samples. While PEF primarily enhances radical scavenging activity and reducing power at lower enzyme concentrations, US pretreatment at 1 % enzyme concentration contributes to favorable effect on DH and hydrolysis efficiency, MW distribution and bioactive peptide formation. However, both technologies demonstrated substantial potential as pretreatment to boost the effectiveness of enzymatic hydrolysis process for generation of bioactive peptides from starfish collagen.

While this study evaluated the individual effects of PEF and US pretreatments on enhancing enzymatic hydrolysis and bioactivity of marine collagen, emerging research suggests that combining non-thermal technologies may offer synergistic advantages. Soltanzadeh et al. [73] emphasized that PEF, when integrated with complementary methods such as ultrasound, can promote greater protein modification, enzyme accessibility, and functional improvements while preserving product quality. These synergistic effects are attributed to the distinct mechanisms of PEF-induced electroporation and US-driven cavitation, which together may enhance substrate permeability and enzymatic efficiency.

## 4 Conclusions

The efficiency of US and PEF as pretreatments in improving the susceptibility of starfish collagen to enzymatic hydrolysis at two enzyme to substrate ratios was evaluated along with in vitro antioxidant capacity of the released collagen peptides. Application of both pretreatments significantly enhanced the DH achieved during subsequent enzymatic hydrolysis compared with the control, and at 1 % v/w enzyme, the US treatment was more effective than PEF. Both US and PEF pre-treatments reduced the time required to reach a specific DH, and also increased the absolute DH at a specific enzyme concentration, leading to higher recovery of SFCB hydrolysates. It was especially important that the pretreaments effectively could compensate for the effect of a low enzyme to substrate ratio (0.5 %v/w). Pretreatment with both PEF and US further resulted in the generation of collagen peptides with significantly higher antioxidant properties compared with the conventional hydrolysis. Applying PEF before the hydrolysis with low enzyme to substrate ratio reduced the IC50 of the generated peptides for ABTS radical inhibition to half compared with the conventional enzymatic hydrolysis. The improvements in the antioxidant activity were explained by the effect of the pretreaments on the molecular weight distribution of the generated peptides and their amino acid compositon. Both pretreatments resulted in an increase in the proportion of LMW peptides (<10 kDa), compared with the conventional method. This increase could be attributed to the ability of the US to induce protein structure relaxation and enhance enzyme-substrate contact, while PEF can facilitate for the latter via protein unfolding. The abundance of hydrophobic amino acid, and basic amino acids which are associated with antioxidative potential, also increased in the SFCB hydrolysates generated after applying US and PEF pretreatment. While PEF primarily enhances radical scavenging activity and reducing power at lower enzyme concentrations, US pretreatment at 1 % enzyme concentration contributes to favorable effect on DH and hydrolysis efficiency, MW distribution and bioactive peptide formation. The results indicate that each pretreatment offers distinct advantages depending on the processing conditions. Altogether, these findings underscore the potential of both PEF and US technologies in increasing collagen susceptibility and improving enzymatic hydrolysis, especially under low enzyme to substrate conditions, and generating peptides with higher antioxidant activity. Although our current work focused on separate applications, future studies should investigate combined or sequential PEF and US treatments for collagen hydrolysis, as this approach might further improve the release of bioactive peptides and enhance antioxidant functionality. Also focus on identifying specific bioactive peptide sequences, understanding secondary structure modifications induced by US and PEF pretreatments, and linking these structural features to observed functional outcomes such as antioxidant capacity.

## CRediT authorship contribution statement

**Elahe Sharifi:** Writing – review & editing, Writing – original draft, Visualization, Software, Methodology, Investigation, Formal analysis, Data curation. **Ingrid Undeland:** Writing – review & editing, Project administration, Funding acquisition. **Mehdi Abdollahi:** Writing – review & editing, Visualization, Validation, Supervision, Resources, Project administration, Funding acquisition, Conceptualization.

## Declaration of competing interest

The authors declare that they have no known competing financial interests or personal relationships that could have appeared to influence the work reported in this paper.
